# Supplementary material for: Multiomic profiling of the acute stress response in the mouse hippocampus
Source: Nat Commun. 2022 Apr 5;13:1824. doi: 10.1038/s41467-022-29367-5 (PMC8983670; doi:10.1038/s41467-022-29367-5)
Supplement: Supplementary file 1 — Supplementary Information [file 41467_2022_29367_MOESM1_ESM.pdf]

Supplementary Information

Multiomic profiling of the acute stress response in the mouse hippocampus

Lukas M. von Ziegler*[1,2], Amalia Floriou-Servou*[1,2], Rebecca Waag[1,2], Rebecca R. Das Gupta[3,4], Oliver Sturman[1,2], Katharina Gapp[1,2], Christina A. Maat[1,2], Tobias Kockmann[5], Han-Yu Lin[1,2,6], Sian N. Duss[1,2], Mattia Privitera[1,2], Laura Hinte[7], Ferdinand von Meyenn[7], Hanns U. Zeilhofer[2,3,4], Pierre-Luc Germain[2,8,9], Johannes Bohacek[1,2#]

[1] Laboratory of Molecular and Behavioral Neuroscience, Institute for Neuroscience, Department of Health Sciences and Technology, ETH Zurich, Switzerland
[2] Neuroscience Center Zurich, ETH Zurich and University of Zurich, Switzerland
[3] Institute of Pharmacology and Toxicology, University of Zurich, Switzerland
[4] Institute of Pharmaceutical Sciences, ETH Zurich, Switzerland
[5] Functional Genomics Center Zurich, ETH Zurich and University of Zurich, Switzerland
[6] Institute of Pharmacology and Toxicology, University of Zurich-Vetsuisse, Zurich, Switzerland
[7] Laboratory of Nutrition and Metabolic Epigenetics, Institute of Food, Nutrition and Health, Department of Health Sciences and Technology, ETH Zurich, Switzerland
[8] Computational Neurogenomics, Institute for Neuroscience, Department of Health Sciences and Technology, ETH Zürich, Switzerland
[9] Laboratory of Statistical Bioinformatics, Department for Molecular Life Sciences, University of Zürich, Switzerland

# Corresponding Author: Johannes Bohacek, johannes.bohacek@hest.ethz.ch

Supplementary Figures

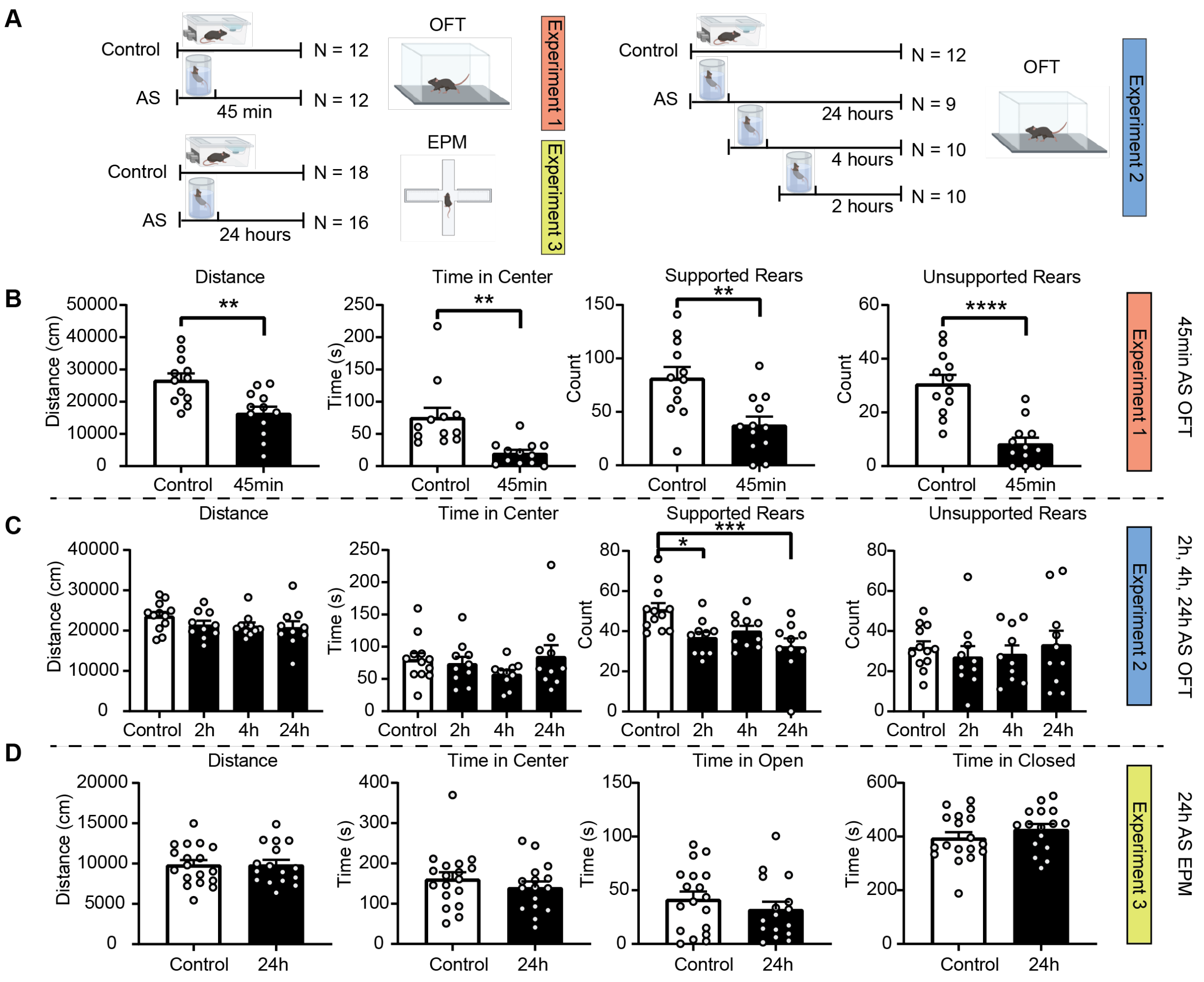

Figure S1: Stress induced changes in behavior across time. A) Overview of 3 different experiments to assess stress induced behavioral changes at different time points. **B)** Stress induced changes in the OFT after 45 mins. Stressed animals show reduced distance traveled (unpaired t-test; t=3.548, df=22, p=0.0018), reduced time in center (two-sided unpaired t-test; t=3.5, df=22, p=0.0020) as well as reduced supported (unpaired t-test; t=3.393, df=22, p=0.0026) and unsupported (two-sided unpaired t-test; t=5.532, df=22, p = 1.47e-05) rearing compared to controls (Ctrl N = 12; 45 min swim N = 12). **C)** Stress induced changes in the OFT after 2h, 4h and 24h. Stressed animals show reduced supported rearing (One-way ANOVA; F(3,38)=6.122, P=0.0017, Dunnett's multiple comparisons test: Control vs 2h: adj. p=0.0115, Control vs 4h: adj. p=0.0007), however no change in distance traveled, time in center and unsupported rearing (Ctrl N=12, 2h swim N=10, 4h swim N=10, 24h swim N=24). *p < 0.05, ** p < 0.01, *** p < 0.001, **** p < 0.0001, bars depict mean ± SEM. Experiments performed in male mice.

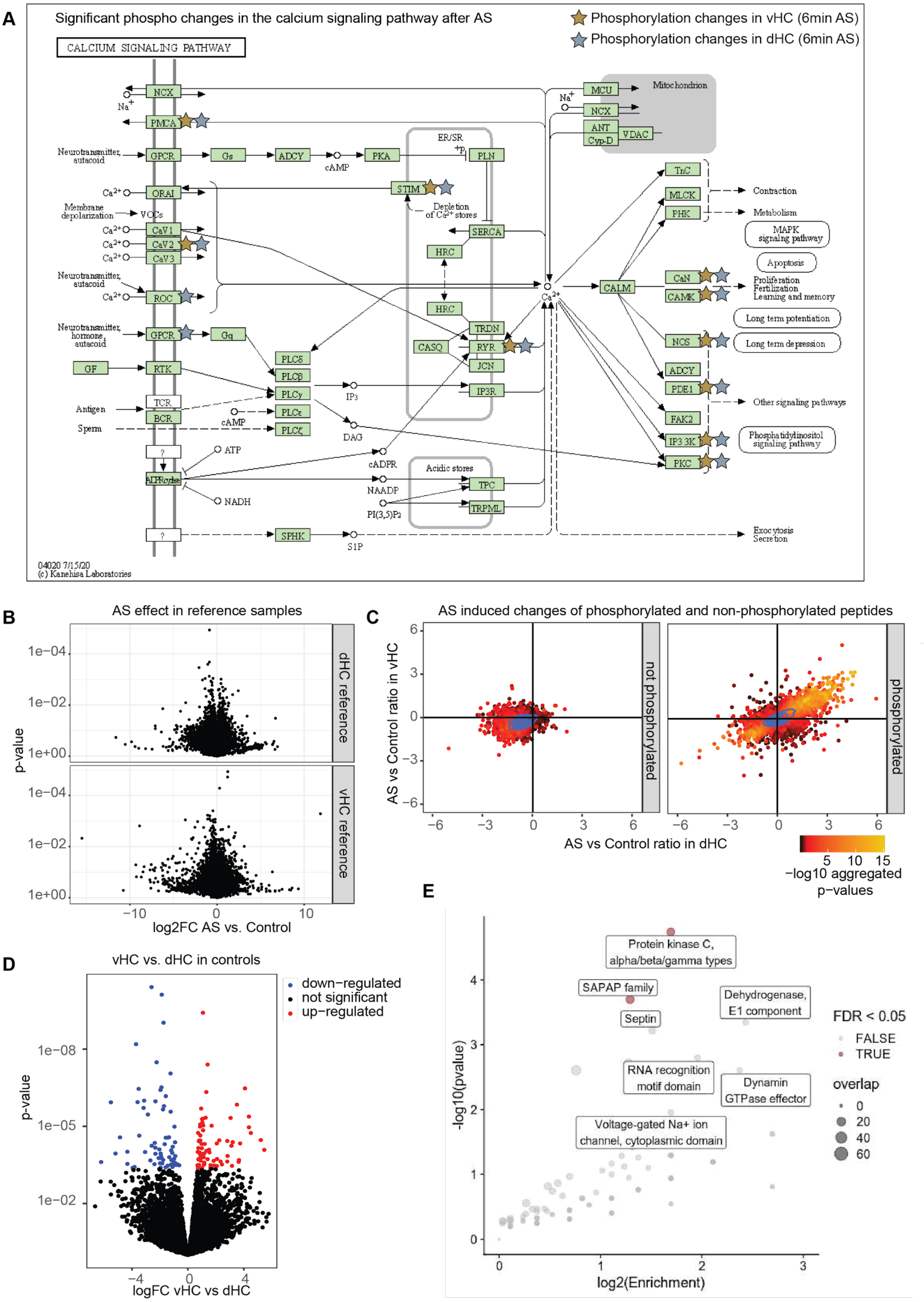

Figure S2: The phosphoproteome of acute swim stress. A) Calcium signaling pathway proteins with altered phosphorylation immediately after stress in vHC and dHC. Red stars label proteins which have upregulated phosphosites after acute stress. Original illustration adapted from kegg pathways (www.genome.jp/kegg). **B)** Both in dHC and vHC no significant changes are observed in reference samples (N = 4 X 4) after multiple testing correction, indicating no stress induced change of total protein expression. **C)** Strength of swim effects (6 min time point) on phosphorylated vs non-phosphorylated peptides in vHC and dHC. **D)** Volcano plots depicting vHC vs dHC baseline differences in the control group. **E)** Over-representation of InterPro protein domains among significantly altered phosphopeptides. Significantly overrepresented domains (FDR < 0.05) are labeled in red.

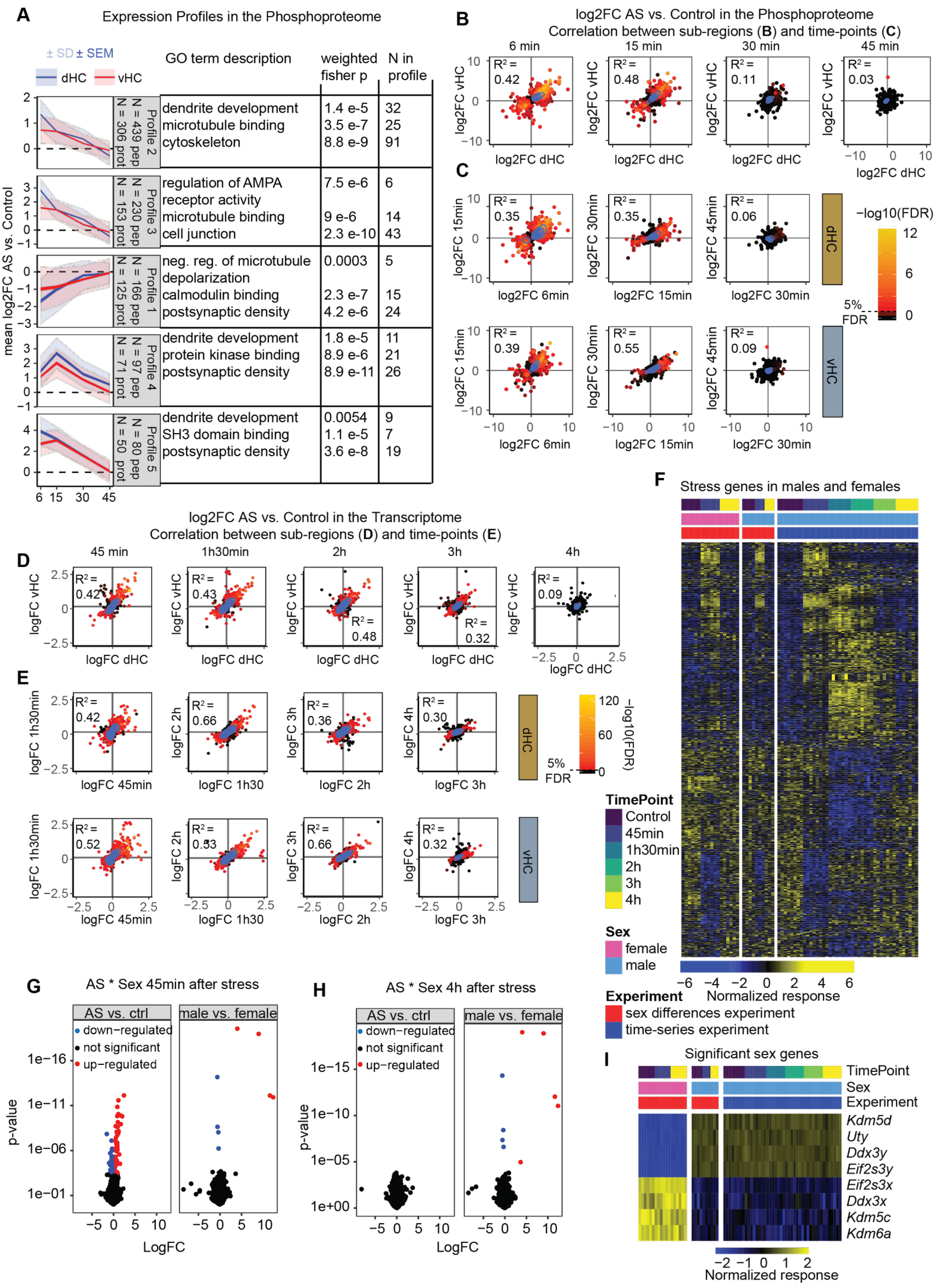

Figure S3: Phosphoproteomic and transcriptomic effects of swim stress on the hippocampus. A) *K*-means clustering of significantly modified peptide changes to resolve 5 temporal profiles, and GO term analysis of the corresponding proteins. Blue indicates expression in dHC, red in vHC. Plots indicate both standard error of the mean (SEM) of logFC in bright color and standard deviation (SD) in faint color. **B)** Scatterplot illustrating modified peptide logFCs of stress vs. control groups in the vHC vs. the dHC at each time point, colored by aggregated significance. (all correlations are significant with $p < 1.18$ e-8) **(C)** Scatterplot illustrating modified

peptide logFCs of stress vs. control groups of successive time points in separate hemispheres, colored by aggregated significance (all correlations are significant with $p < 1.11$ e-22). **D)** Scatterplot illustrating mRNA logFCs of stress vs. control groups in the vHC vs. the dHC at each time point, colored by aggregated significance (all correlations are significant with $p < 4.8$ e-19). Ceilings for logFC set to 2.5. **(E)** Scatterplot illustrating mRNA logFCs of stress vs. control groups of successive time points in separate hemispheres, colored by aggregated significance (all correlations are significant with $p < 2.5$ e-70). Ceilings for logFC set to 2.5. **F)** Stress responsive genes from the bulk sequencing experiment in male and female vHC at 45 mins and 4 hours. **G)** Volcanoplots depicting statistical results of stress and sex effects at 45 mins in the vHC transcriptome. **H)** Volcanoplots depicting statistical results of stress and sex effects at 4 hours in the vHC transcriptome. **I)** Heatmap of significant sex genes.

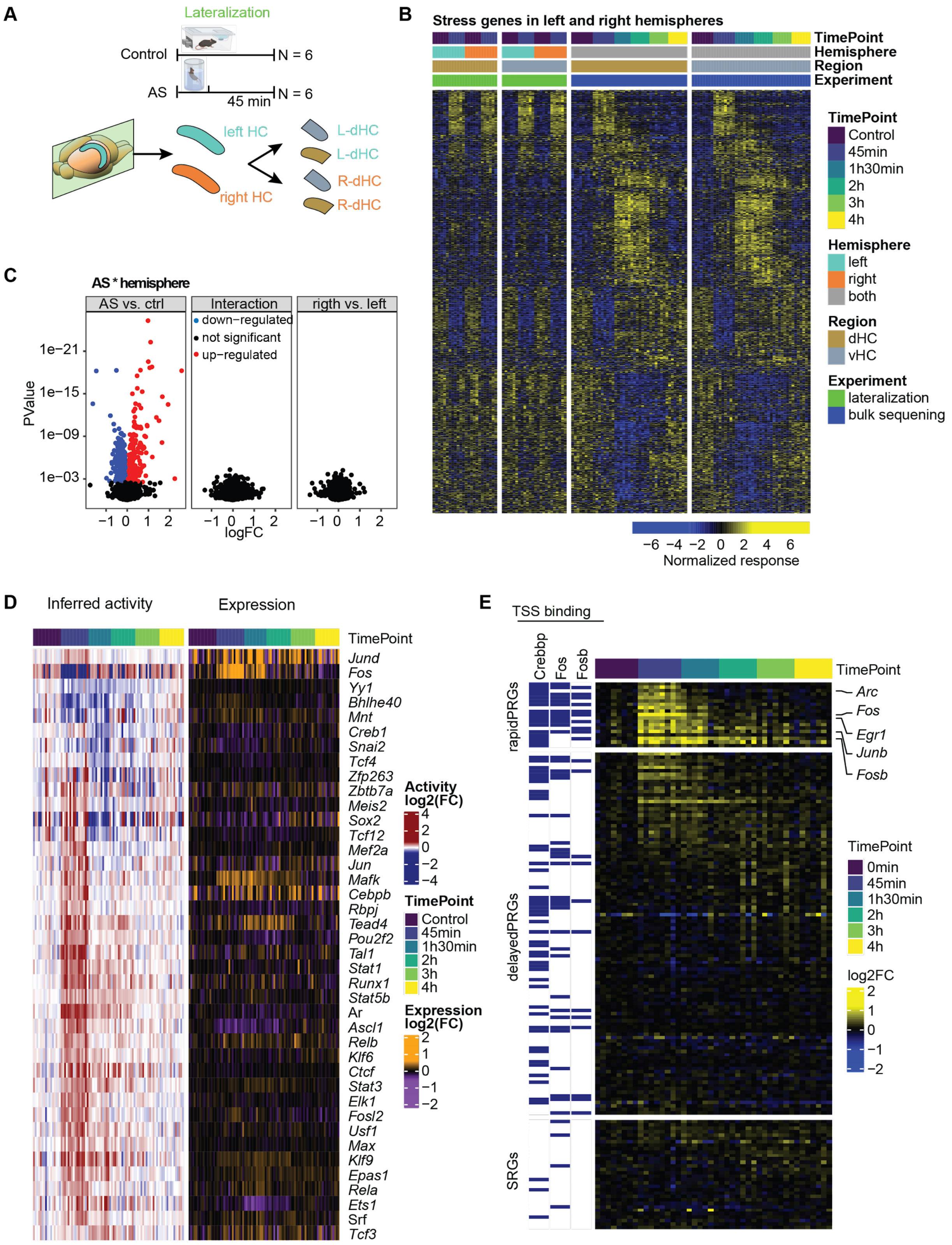

Figure S4: Transcription factor analyses and transcriptomics effects of swim stress and in the left and right hemispheres. A) Experimental design of the lateralization experiment. **B)** Stress responsive genes from the bulk sequencing experiment left and right hemispheres. **C)** Volcano plots depicting statistical results of stress and hemisphere effects and hemisphere:stress interactions at 45 mins. **D)** Relative inferred activity (left) and expression (right) of the transcription factors showing significant changes in activity. **E)** Expression of known activity-responsive genes [50] upon AS. The left shows whether the genes have a binding site for key TFs at their TSS, based on ChIP-seq from neuronal cultures [68,124]. Experiments performed with male mice.

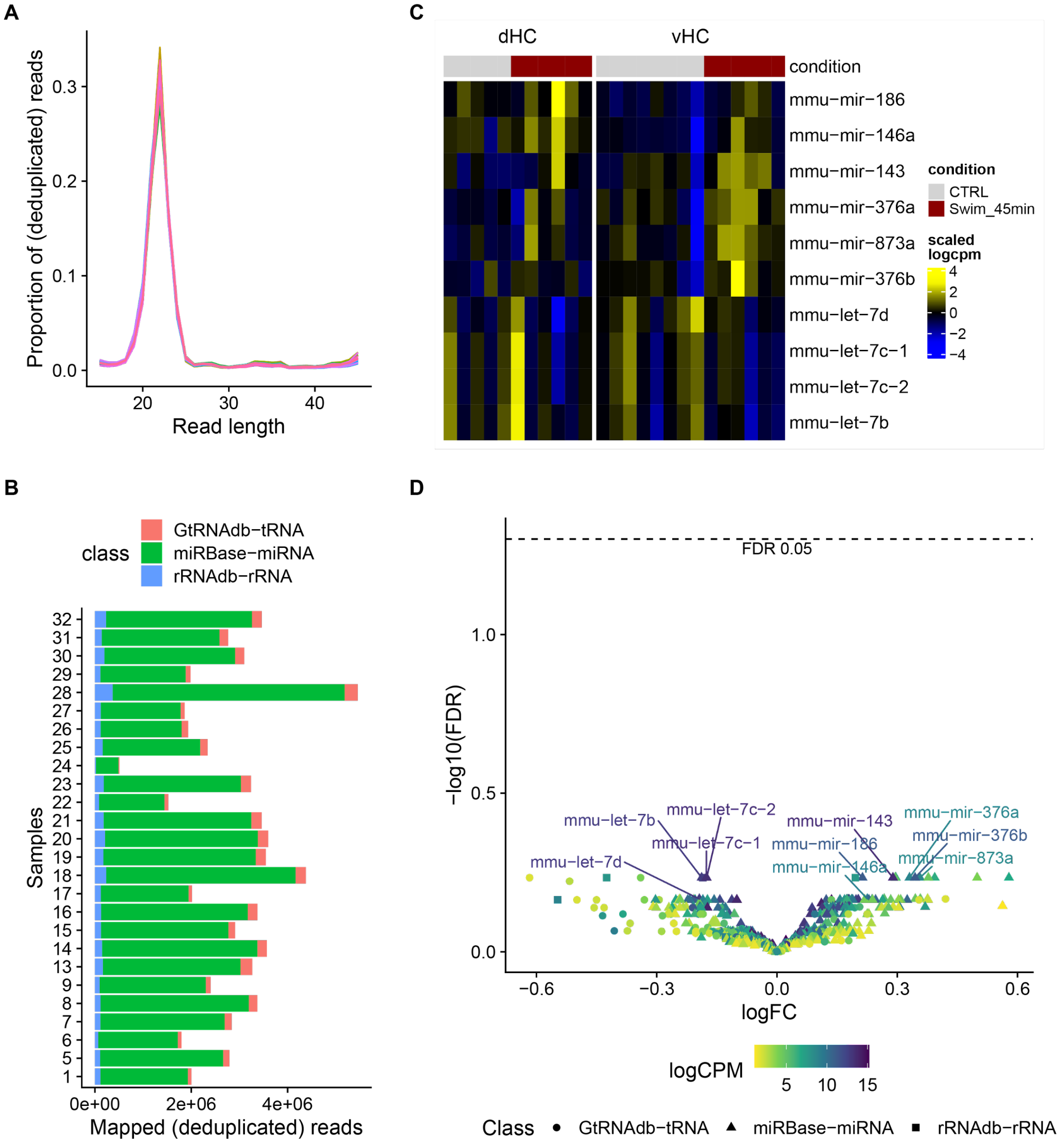

Figure S5: miRNA sequencing. A) Read length distribution across samples after UMI and adapter trimming. **B)** Mapped (deduplicated) reads assigned to the major small RNA classes. Sample 24 was excluded from downstream analysis due to low coverage. **C)** Relative expression profile of the top candidate miRNAs (not passing genome-wide significance). **D)** Volcano plot of the differential expression results. Experiments performed with male mice.

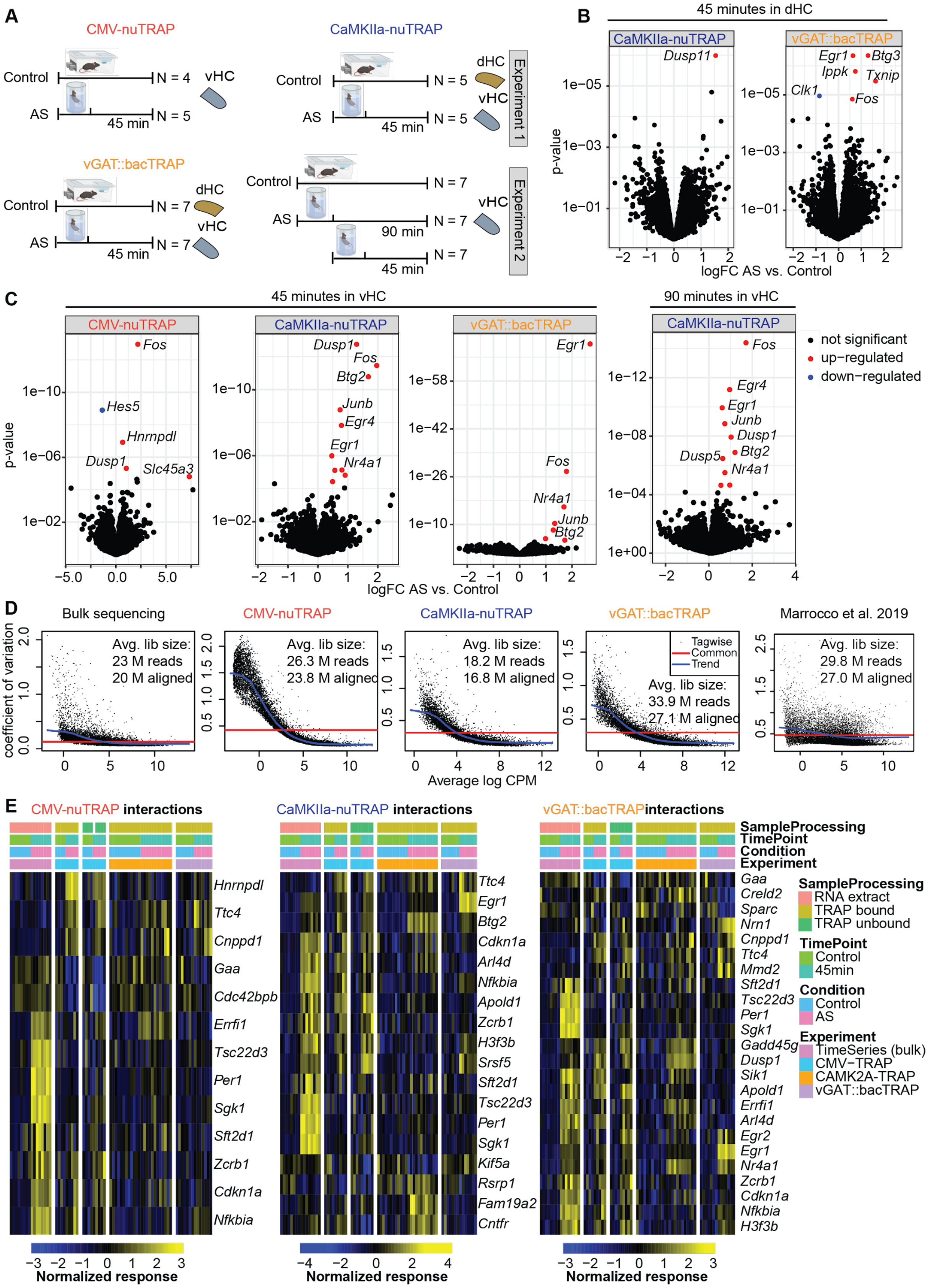

Figure S6: Effects of swim stress on the whole translatome and the translatome of inhibitory and excitatory neurons. A) Experimental design of all TRAP experiments. **B)** Volcano plots depicting significant changes in translation in excitatory neurons (CaMKIIa-nuTRAP) and inhibitory neurons (vGAT::bacTRAP) in the dHC at 45 mins. **C)** Volcano plots depicting significant changes in translation in whole tissue (CMV-nuTRAP), excitatory neurons (CaMKIIa-nuTRAP) and inhibitory neurons (vGAT::bacTRAP) in the vHC at 45 mins and in excitatory neurons (CaMKIIa-nuTRAP) at 90 mins. **D)** Plots depicting coefficient of variation (CV) vs logCPM in bulk sequencing, CMV-nuTRAP, CaMKIIa-nuTRAP and vGAT::bacTRAP in the vHC at 45 mins swim vs. controls. CV of Marrocco et al. 2019 is shown as comparison to published TRAP data (CA3 neurons). **E)** Heatmaps depicting DEGs with significant interaction terms (p interaction <= 0.05) in CMV-nuTRAP, CaMKIIa-nuTRAP and vGAT::bacTRAP and their expression across all datasets. Experiments performed with males (Camk2a-NuTRAP and vGAT::backTRAP) and females (CMV-nuTRAP).

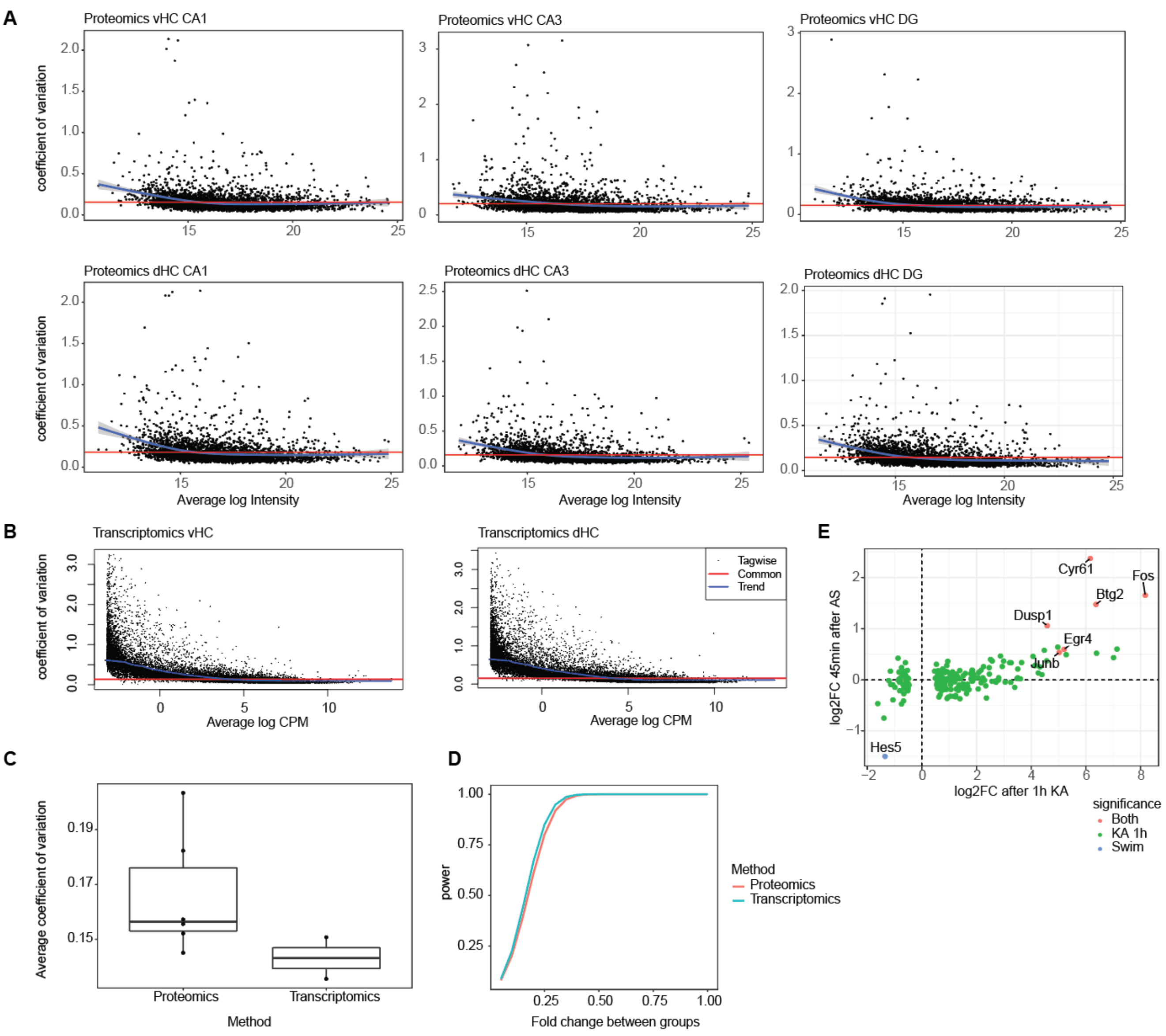

Figure S7: Variability and power of proteomics and transcriptomics analyses. A) coefficient of variation (CV) in individual regions and sub-regions using proteomics. **B)** CV in individual regions using transcriptomics. **C)** Mean CV values from A/B shown as boxplot. Proteomics: minima = 0.145, maxima = 0.2, centre 0.156, bounds of box: upper = 0.176, lower = 0.153; Transcriptomics: minima = 0.136, maxima = 0.15, centre 0.143, bounds of box: upper = 0.147, lower = 0.14. No significant difference with two-sample Wilcoxon signed rank test ($W(6,2) = 11$, $p = 0.1429$) **D)** Power analysis demonstrating similar sensitivity for both methods in respect to the fold change between groups (calculated with $\alpha = 0.05$; N = 7 / CV = 0.143 for transcriptomics and N = 8 / CV = 0.166 for proteomics). **E)** Comparison of the changes 45 mins after acute stress (y axis, this study) with the changes after one hour of sustained kainate exposure (x axis, see ref. 68), both in hippocampal Camk2a-TRAP.
