# Supplementary material for: Multiomic profiling of the acute stress response in the mouse hippocampus
Source: Nat Commun. 2022 Apr 5;13:1824. doi: 10.1038/s41467-022-29367-5 (PMC8983670; doi:10.1038/s41467-022-29367-5)
Supplement: Supplementary file 2 — Reporting Summary [file 41467_2022_29367_MOESM2_ESM.pdf]

nature research

Corresponding author(s): Johannes Bohacek

Last updated by author(s): Dec 1, 2021

Reporting Summary

Nature Research wishes to improve the reproducibility of the work that we publish. This form provides structure for consistency and transparency in reporting. For further information on Nature Research policies, see our Editorial Policies and the Editorial Policy Checklist.

Statistics

For all statistical analyses, confirm that the following items are present in the figure legend, table legend, main text, or Methods section.

n/a	Confirmed	
☐	☒	The exact sample size (n) for each experimental group/condition, given as a discrete number and unit of measurement
☐	☒	A statement on whether measurements were taken from distinct samples or whether the same sample was measured repeatedly
☐	☒	The statistical test(s) used AND whether they are one- or two-sided<br>*Only common tests should be described solely by name; describe more complex techniques in the Methods section.*
☐	☒	A description of all covariates tested
☐	☒	A description of any assumptions or corrections, such as tests of normality and adjustment for multiple comparisons
☐	☒	A full description of the statistical parameters including central tendency (e.g. means) or other basic estimates (e.g. regression coefficient) AND variation (e.g. standard deviation) or associated estimates of uncertainty (e.g. confidence intervals)
☐	☒	For null hypothesis testing, the test statistic (e.g. F, t, r) with confidence intervals, effect sizes, degrees of freedom and P value noted<br>*Give P values as exact values whenever suitable.*
☒	☐	For Bayesian analysis, information on the choice of priors and Markov chain Monte Carlo settings
☐	☒	For hierarchical and complex designs, identification of the appropriate level for tests and full reporting of outcomes
☐	☒	Estimates of effect sizes (e.g. Cohen's d, Pearson's r), indicating how they were calculated

Our web collection on statistics for biologists contains articles on many of the points above.

Software and code

Policy information about availability of computer code

Data collection	Xcalibur (v.4.3)
Data analysis	Standalone softwares:<br>Spectronaut (version 10 and version 11 (phosphoproteome males vs female))<br>cutadapt (v. 1.15)<br>kallisto (v. 0.44.0)<br>STAR (v.2.7.3a)<br>featureCounts (v1.6.4)<br>Cell Ranger (v. 4.0.0)<br>Sports (v.1.0.0)<br>RStudio (v.1.3.1073) using R (v.3.6.3)<br><br>Analysis packages used with R:<br>biganalytics (v.1.1.21)<br>M3C (v.1.8.0)<br>DEP (v.1.6.1)<br>edgeR (v 3.26.8)<br>sva (v.3.34.0)<br>SoupX (v.1.4.5)<br>scDblFinder (v.1.4.0)<br>scater (v.1.18.0)<br>sctransform (v.0.3.1)

Seurat (v.3.2.2)
SETools(v.1.8.0)
scClassify (v.1.2.0)
muscat (v.1.4.0)
limma (v.3.46)
goseq (v.1.41)
viper (v.1.24)

all custom code has been publicly deposited online under:
https://github.com/ETHZ-INS/StressomeExplorer

For manuscripts utilizing custom algorithms or software that are central to the research but not yet described in published literature, software must be made available to editors and reviewers. We strongly encourage code deposition in a community repository (e.g. GitHub). See the Nature Research guidelines for submitting code & software for further information.

Data

Policy information about availability of data

All manuscripts must include a data availability statement. This statement should provide the following information, where applicable:

- Accession codes, unique identifiers, or web links for publicly available datasets
- A list of figures that have associated raw data
- A description of any restrictions on data availability

All LC-MS/MS data are available to readers via ProteomeXchange with identifier PXD024829 and PXD029903. All sequencing data are available to readers via Gene Expression Omnibus with identifiers GSE169505 (bulk and TRAP sequencing), GSE169509 (smallRNA sequencing) and GSE169510 (snRNA sequencing). Code for the interactive web app and all analyses (independent scripts) can be found at https://github.com/ETHZ-INS/StressomeExplorer. Selected statistical results showing group contrasts and gene/protein clusters are reported in Table S1 (phosphoproteomics), Table S2 (transcriptomics) and Table S3 (proteomics).

Raw data associated with figures:
Figure 1: Proteome data (ProteomeXchange ID PXD024829 and ProteomeXchange ID PXD029903)
Figure 2: Sequencing data (GEO ID GSE169505)
Figure 3: Sequencing data (GEO ID GSE169505)
Figure 4: Sequencing data (GEO ID GSE169510)
Figure 5: Sequencing data (GEO ID GSE169510)
Figure 6: Sequencing data (GEO ID GSE169505)
Figure 7: Proteome data (ProteomeXchange ID PXD024829)
Figure S2: Proteome data (ProteomeXchange ID PXD024829)
Figure S3: Proteome data (ProteomeXchange ID PXD029903) and Sequencing data (GEO ID GSE169505)
Figure S4: Sequencing data (GEO ID GSE169505)
Figure S5: Sequencing data (GEO ID GSE169509)
Figure S6: Sequencing data (GEO ID GSE169505)
Figure S7: Proteome data (ProteomeXchange ID PXD024829) and Sequencing data (GEO ID GSE169505)

Field-specific reporting

Please select the one below that is the best fit for your research. If you are not sure, read the appropriate sections before making your selection.

☒ Life sciences ☐ Behavioural & social sciences ☐ Ecological, evolutionary & environmental sciences

For a reference copy of the document with all sections, see nature.com/documents/nr-reporting-summary-flat.pdf

Life sciences study design

All studies must disclose on these points even when the disclosure is negative.

Sample size

Sample sizes vary between different experiments and are listed in the manuscript in figures and legends.
For behavior sample sizes of ~N = 9-18 / group were used
for phosphoproteomic / proteomic experiments sample sizes of N = 5-8 / group were used
for trancriptomic experiments sample sizes of N = 5-8 / group were used
for snRNA sequencing sample sizes of N = 2 / group were used
No statistical methods were used to determine sample size. We used experience from previous published studies that our lab conducted to estimate the appropriate number of replicates for behavioral, proteomic and transcriptomic experiments to reliably detect moderate expression/phenotype changes. For snRNA sequencing sample size was limited due to technical and financial aspects.

Data exclusions

For Figure 1 a single animal was excluded due to strong behavioral abnormality at both OFT tests (the animal was sitting in the center of the arena the entire time). No other data was excluded across all experiments

Replication

All attempts of replication were successful. More specifically:
Experiments of Figure 1 (phosphoproteomic) were replicated with a second experiment (time-series phosphoproteomics) and selected phosphorylations were validated using western blot (Figure 1F)

	For Figure 2 B (Time series bulk sequencing), replication of the 45 minutes time-point were conducted in both hemispheres (Figure S4 B/C) and males / females (Figure 2 G / I).<br>For TRAP sequencing the 45minutes time-point of Camk2A mice was repeated in a second experiment (See Figure S6A)
Randomization	We used a block design for behavioral experiments, sample processing and measurements to ensure proper randomization. In brief animals / samples were split into multiple blocks, containing one replicate of each condition. Processing order within these blocks was randomized, as was the order of blocks in the experiment. Full detail about behavior/processing/measurement order can be provided.
Blinding	Investigators were blinded during behavior and sample processing, but not during the analysis process. However, the same algorithmic analysis methods were used for all samples within each experiment

Reporting for specific materials, systems and methods

We require information from authors about some types of materials, experimental systems and methods used in many studies. Here, indicate whether each material, system or method listed is relevant to your study. If you are not sure if a list item applies to your research, read the appropriate section before selecting a response.

Materials & experimental systems

n/a	Involved in the study	
☐	☒	Antibodies
☒	☐	Eukaryotic cell lines
☒	☐	Palaeontology and archaeology
☐	☒	Animals and other organisms
☒	☐	Human research participants
☒	☐	Clinical data
☒	☐	Dual use research of concern

Methods

n/a	Involved in the study	
☒	☐	ChIP-seq
☒	☐	Flow cytometry
☒	☐	MRI-based neuroimaging

Antibodies

Antibodies used	Antibodies for immunopurification during TRAP: GFP antibodies (Memorial Sloan-Kettering Monoclonal Antibody Facility; clone names: Htz-GFP-19F7 and Htz-GFP-19C8, bioreactor supernatant purity)<br>Immunohistochemistry antibodies: chicken anti-GFP (ab13970, Abcam, polyclonal), chicken anti-GFP (GFP-1020, Aves, polyclonal), rabbit anti-mCherry (ab167453, Abcam, polyclonal), rabbit anti-GAD65/67 (AB1511, Millipore, polyclonal), rabbit anti-NeuN (ab177487, Abcam, clone name: EPR12763), mouse anti-GFAP (G3893, Sigma-Aldrich, clone name: G-A-5), goat anti-chicken Alexa Fluor 488 (A-11039, Thermo Fischer Scientific, polyclonal), goat anti-rabbit Alexa 546 (A11035, Thermo Fisher Scientific, polyclonal), goat anti-mouse Cy™3 (115-165-003, Jackson ImmunoResearch, polyclonal), goat anti-rabbit Alexa Fluor Plus 647 (A32733, Invitrogen, polyclonal).<br>Western blot antibodies: anti-pS553-Syn1 rabbit (ab32532, Abcam, clone name: E377) and anti-GAPDH rabbit (ABS16, Millipore, polyclonal), goat anti-rabbit IRDye800CW (926-32211, LI-COR Biosciences GmbH).
Validation	Antibodies for immunopurification during TRAP were chosen based on the publication of Heiman et al., 2014.<br>All primary antibodies used for immunohistochemistry and Western blotting were validated by the manufacturers by comparison to positive and negative controls, and additionally tested in our lab.<br>See manufacturers documentation for positive and negative controls [=URL]:<br>chicken anti-GFP (ab13970, Abcam) [https://www.abcam.com/gfp-antibody-ab13970.html]<br>chicken anti-GFP (GFP-1020, Aves) [https://www.aveslabs.com/products/anti-green-fluorescent-protein-antibody-gfp]<br>rabbit anti-mCherry (ab167453, Abcam) [https://www.abcam.com/mcherry-antibody-ab167453.html]<br>rabbit anti-GAD65/67 (AB1511, Millipore) [https://www.merckmillipore.com/CH/de/product/Anti-Glutamate-Decarboxylase-65-67-Antibody,MM_NF-AB1511]<br>rabbit anti-NeuN (EPR12763, ab177487, Abcam) [https://www.abcam.com/neun-antibody-epr12763-neuronal-marker-ab177487.html]<br>mouse anti-GFAP (G3893, Sigma-Aldrich) [https://www.sigmaaldrich.com/CH/de/product/sigma/g3893]<br>anti-pS553-Syn1 rabbit monoclonal (ab32532, Abcam) [https://www.abcam.com/synapsin-i-phospho-s553-antibody-e377-ab32532.html]<br>anti-GAPDH rabbit polyclonal (ABS16, Millipore) [https://www.merckmillipore.com/CH/de/product/Anti-GAPDH-Antibody,MM_NF-ABS16]

Animals and other organisms

Policy information about studies involving animals; ARRIVE guidelines recommended for reporting animal research

Laboratory animals	The experimental animals used in this study belong to the species Mus musculus. The mice were either wild type, strain C57Bl/6J or had one of the following genotypes: CMV-nuTRAP generated by breeding CMV-Cre mice (B6.C-Tg(CMV-cre)1Cgn/J, MGI:3613618) with homozygous or heterozygous floxed nuTRAP mice (B6;129S6-Gt(ROSA)26Sortm2(CAG-NuTRAP)Evdr/J, MGI:104735). CaMKIIa-nuTRAP generated by breeding homozygous floxed nuTRAP mice (B6;129S6-Gt(ROSA)26Sortm2(CAG-NuTRAP)Evdr/J, MGI:104735) with homozygous CaMKIIA-Cre (B6.Cg-Tg(Camk2a-cre)T29-1Stl/J, MGI:3613616) mice. vGAT::bacTRAP (Tg(Slc32a1-RPL10a-eGFP)Uze)

	(Das Gupta et al., 2021).<br>Experiments were conducted with either male or female mice.<br>Mice were housed in groups of 4-5 per cage in a temperature- and humidity-controlled facility on a 12-hour reversed light-dark cycle (lights off: 9:15 am; lights on: 9:15 pm), with food and water ad libitum, and used for experiments at the age of 2-5 months. All experiments were conducted during the animals' active (dark) phase
Wild animals	The study did not involve wild animals.
Field-collected samples	The study did not involve samples collected from the field.
Ethics oversight	All experiments were conducted in accordance with the Swiss federal guidelines for the use of animals in research and under license ZH161/17, approved by the Zurich Cantonal veterinary office.

Note that full information on the approval of the study protocol must also be provided in the manuscript.
