# Supplementary material for: Multiomic profiling of the acute stress response in the mouse hippocampus
Source: Nat Commun. 2022 Apr 5;13:1824. doi: 10.1038/s41467-022-29367-5 (PMC8983670; doi:10.1038/s41467-022-29367-5)
Supplement: Supplementary file 4 — Description of Additional Supplementary Files [file 41467_2022_29367_MOESM4_ESM.pdf]

Title: Supplementary Data 1

Description: This Document contains phosphoproteomics results

Sheet 'Stats'

This sheet contains statistical results from all contrasts Control vs Stress at the different time points for modified peptides. The sheet is in a wide data format, with each column indicating p-values, logFC or FDR values of one contrast. Information about the contrasts can be obtained from the column name. i.e Exp1_dHC_SW06_vs_Exp1_dHC_CON_p.val = p.value of Control vs. Swim at the 6min time point in experiment 1. Further, this sheet contains information about the modified peptide. The column Position indicates potential phosphosites within the modified peptide, the column Probabilities their respective probability.

Sheet 'Clusters'

This sheet contains information about expression profiles (only for significantly changing peptides). The sheet is in a long data format (i.e. each peptide has rows, one for each time-point and in both regions). Cluster indicates the cluster number the peptide was associated with after k-means. Log2FC indicates the fold change of this peptide at the associated time-point and in the associated region

Title: Supplementary Data 2

Description: This Document contains transcriptomic results

Sheet 'Stats'

This sheet contains statistical results from all contrasts Control vs Stress at the different time points for genes. The sheet is in a long data format. Each gene has multiple rows, one for each contrast (i.e one in vHC at 45min, one in vHC at 1h30 min and so on). The columns contain statiatical values (p value, logFC, CPM and adjusted p.value)

Sheet 'Clusters'

This sheet contains information about expression profiles (only for significantly changing genes). The sheet is in a long data format (i.e. each genes has multiple rows, one for each time-point and in both regions). Cluster indicates the cluster number the genes was associated with after k-means. Log2FC inidcates the fold change of this genes at the associated time-point and in the associated region

Sheet 'ProcessedvsUnprocessed'

This sheet contains information about expression of processed (spliced) vs unprocessed (unspliced) transcripts for a given gene. The sheet is in a wide data format. There is one single row for each gene with the column names indicateing the expression of a type (spliced or unspliced) at each time-point. Data of dHC and vHC are aggregated. Example of column names and meaning: logFC.TimePoint3h = logFC of the processed transcript in the contrast swim 3h vs control logFC.typeunspliced. TimePoint3h = logFC of the unprocessed transcript in the contrast swim 3h vs control

Title: Supplementary Data 3

Description: This Document contains proteomic results

Sheet 'Contrasts'

This sheet contains statistical results from all contrasts Control vs Stress in the different regions and subregions 4h following stress. The sheet is in a long data format. Each protein has multiple rows, one for each contrast (i.e one for vHC DG, one for dHC CA1 and so on). The columns contain statiatical values (Pvalue, Log2FC, and adjusted p.value (fdr))

Sheet 'RegionxSubregion'

This sheet contains statistical results from a model that resolves regional and sub-regional differences for pooled groups. The sheet is in a wide data format. Each protein has one single row and the column names contain information about the coefficient tested. There are 3 p.value columns, one for each coefficient (i.e Regional effects = pvalue.Region, interactions between Region and Sub-Region = pvalue.Interaction and so on). There are 5 logFC columns. One for regional effects (log2FC.Region) and three for contrasts between SubRegions (i.e log2FC.CA1CA3 = Contrast between CA1 vs CA3) and the meanLog2FC (meanSubRegionLogFC) between Subregions. there are 3 adjusted p.value columns. They correspond to the p.values after BH (FDR) correction
